# Perceived and actual fighting ability: determinants of success by decision, knockout or submission in human combat sports

**DOI:** 10.1098/rsbl.2020.0443

**Published:** 2020-10-28

**Authors:** Sarah M. Lane, Mark Briffa

**Affiliations:** School of Biological and Marine Sciences, Animal Behaviour Research Group, University of Plymouth, Plymouth, Devon PL4 8AA, UK

**Keywords:** animal contests, mixed martial arts, resource holding potential, skill, vigour

## Abstract

Animal contest theory assumes individuals to possess accurate information about their own fighting ability or resource-holding potential (RHP) and, under some models, that of their opponent. However, owing to the difficulty of disentangling perceived and actual RHP in animals, how accurately individuals are able to assess RHP remains relatively unknown. Furthermore, it is not just individuals within a fight that evaluate RHP. Third-party observers evaluate the fight performance of conspecifics in order to make behavioural decisions. In human combat sports, when fights remain unresolved at the end of the allotted time, bystanders take a more active role, with judges assigning victory based on their assessment of each fighter's performance. Here, we use fight data from mixed martial arts in order to investigate whether perceived fighting performance (judges' decisions) and actual fighting success (fights ending in knockout or submission) are based on the same performance traits, specifically striking skill and vigour. Our results indicate that both performance traits are important for victory, but that vigour is more important for fights resolved via decision, even though the effect of vigour is enhanced by skill. These results suggest that while similar traits are important for fighting success across the board, vigour is overvalued in judges' perceptions of RHP.

## 1. Introduction

The importance of performance traits in determining fighting success has become a major topic of research in the field of animal contest behaviour, with a recent focus on the relative importance of vigour and skill [1,2]. Skill is defined as performing a challenging behaviour well [3], while vigour describes the rate at which a behaviour is performed [3]. Evidence for the role of skill, and its complex association with vigour, in determining success in animal contests has only recently begun to emerge [2,4], but skill has long been recognized in the field of sports science as an important determinant of victory (e.g. [5,6]).

Animal contest theory is predicated on the idea that strategic decisions (such as ‘giving up') are based on a fighter's assessment of their own fighting ability or resource-holding potential (RHP) and that of their opponent. Given the importance of assessment, we should expect selection for accurate perception of RHP. Indeed, theoretical models of fighting behaviour typically imply that contestants should have accurate information about their own RHP and performance [7], with some models further assuming that individuals should also be able to assess their opponent's RHP and that fights should escalate until an accurate assessment can be made [8]. Despite these core assumptions, very little is known about the accuracy with which fighting animals judge the abilities of their rivals, likely owing to the difficulty of disentangling perceived and actual RHP. In the case of animal contests, where it is not possible to ‘ask' an individual how it rates its rival, any insights rely on motivational probing in which fights are interrupted and latency to re-engage is measured [9,10]. Accuracy of RHP assessment can also be important beyond the opponents directly engaged in the fight. Bystanders often observe and evaluate fighters' performances in order to choose future mating partners [3,11–13] or learn which rivals to avoid [14,15]. In human combat sports, third-party observers take on a more active role, whereby a panel of judges assigns victory based on their assessment of each fighter's performance. Thus, in both animal and human contests, perceptions of RHP can drive outcomes and inform critical decisions.

Animal fights generally end when one opponent decides to submit and retreat [16]. Assuming both fighters are equally motivated, the individual of lower RHP will reach this point first and thus lose. This decision can occur early on before escalation to a physical fight, or as a result of energetic and/or damage costs accumulated over time [17–19]. Thus, in some fights, losers decide to quit even though they have the capacity to continue for longer, while in other, rarer examples losers are forced to quit owing to constraints on effective fighting performance. Similarly, in human combat sports such as mixed martial arts (MMA), victory can occur through the direct effect of the winner's actions on the loser, resulting in either (i) a submission (an athlete taps out or verbally concedes the fight) or (ii) a knockout (KO)/technical knockout (TKO) (an athlete is knocked unconscious by their opponent (KO) or unable to continue owing to injuries incurred (TKO)). However, in contrast with animal contests, victory in sports such as MMA can be determined in a third way, via the assessment of a panel of judges. In MMA, if neither fighter elicits a KO/TKO or submission by the end of the timed rounds, the winner is decided using judges' scores collated across the rounds.

The assumption is that the judges' decision accurately reflects the relative performance of each fighter (and thus their relative RHPs), meaning that the judges' ruling should be analogous to the decision the loser would eventually make for themselves if the fight were to continue. If the judges' decision is truly analogous to the loser's decision then similar components of fighting ability should differentiate winners from losers in fights that are decided by a panel of judges and fights that are resolved by direct constraints (e.g. KO or submission), in particular via submission. However, despite following a specific set of criteria (under the unified rules of MMA [20]), judges do not always agree on scores, as evidenced by the occurrence of split and majority decisions, suggesting that there is a degree of error inherent in judging fighting performance. Differences in human perceptions of behaviour are widely appreciated in scientific research, driving the need for blind studies and single observers [21–23]. Human combat sports provide a unique scenario in which to explore the relationship between perceived and actual RHP in terms of performance traits such as skill and vigour.

Research into combat sports, and in particular MMA, is largely focused on data from men's fights, despite there being more than 100 elite female MMA fighters. Similarly, in the field of animal contest research, most studies focus on male–male contests, but studies that have examined female fights often show stark differences in the agonistic behaviours exhibited by males and females. For instance, females of multiple species have been shown to be more aggressive than males, forgoing ritualized displays (e.g. jumping spiders [24]) and attacking more readily than their male counterparts (e.g. anole lizards [25] and convict cichlids [26]). Furthermore, sex differences in performance traits have been seen in hermit crabs *Pagurus bernhardus* with females demonstrating higher levels of vigour [27].

Here, using freely available data on MMA fights, we investigate (i) whether the same measures of fight performance (skill and vigour) predict success in fights ended by decision (perceived fighting performance) and those ending via actual defeat (KOs/submissions), and (ii) whether the importance of these traits for success under both types of resolution differs for male and female fights.

## 2. Material and methods

MMA fight data were collated from UFCstats.com (a database that provides the official statistics for the Ultimate Fighting Championship (UFC)) for all completed fights listed from February 2019 to March 2020 (*N* = 548 fights; women's = 102, men's = 446). These fights involved 599 different fighters (110 women, 489 men), who fought an average of 1.83 times each (range = 1–6 fights per athlete). For the purposes of this study, the following data were collated from each fight: (1) per cent significant strikes landed—described as a measure of accuracy by UFC and used in our analyses as our measure of this component of skill (as defined in [1]); (2) number of strikes attempted per second (calculated as the total number of strikes attempted divided by fight duration), our measure of vigour (as defined in [1]); (3) outcome (win or lose); (4) method of resolution—outcome decided by the judges' scored assessments (hereafter ‘decision'), as a result of a knockout or technical knockout (hereafter ‘KO/TKO') (UFC does not discriminate between knockouts and technical knockouts on ufcstats.com) or a submission. In order to analyse the method of resolution at different levels, for half of the analyses KO/TKO and submission were grouped together as fights ending in ‘defeat'; (5) sex (male or female); (6) fighter ID.

As the levels of skill and vigour expressed by one fighter are likely dependent on the behaviour of their opponent, we only used data from one ‘focal' individual per fight, treating ‘fight' as the level of replication. ‘Focal fighters' were assigned at random by alternating between red and blue fighters (this also avoided any confounding effect of fighter colour on outcome—an effect known to exist in other sports (e.g. [28,29])). We then used generalized linear mixed effects models (GLMMs) with a binomial error family to analyse the effect of focal per cent significant strikes (skill), focal number of strikes per second (vigour), method of resolution (decision, KO/TKO or submission), sex and their interactions on a focal outcome. Random intercepts were included to account for the ID of both athletes (red and blue) per fight as individuals appeared multiple times in the dataset. Two models were run, the first in which method of resolution was split as decision or defeat (KO/TKO and submission grouped together) and the second in which all methods of resolution were incorporated (decision, KO/TKO and submission; TKO via doctor's stoppage was not included as only five fights ended this way). This allowed us to compare outcomes determined through bystander decision and actual defeat and then to separate out the effects of specific methods of resolution. Owing to their very different scales, the strike metrics were standardized (using the calculation *x*-mean/s.d.) prior to analysis to aid model convergence. There was no evidence of overdispersion, nor any strong pattern in the residuals. We conducted model simplification via backwards elimination to remove non-significant terms, determining statistical significance using log-likelihood ratio tests. The results presented here are from the minimal adequate model. All analyses were carried out in RStudio (v. 1.1.456 [30]) using the package lme4 [31]; R code used in this analysis is available in the electronic supplementary material.

## 3. Results

### (a) Decision versus defeat

**Figure 1.**
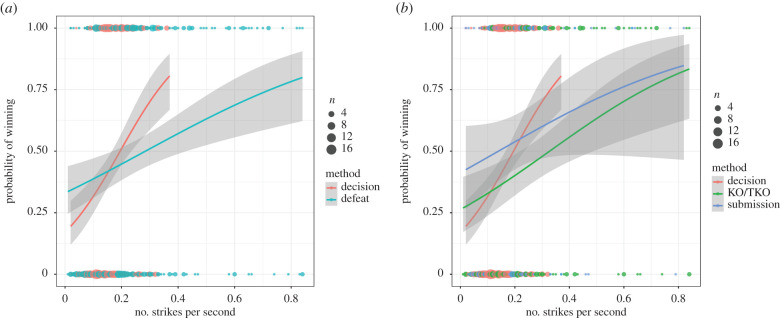
Interaction between the number of strikes attempted per second (vigour) and the method of resolution on the likelihood of winning. (*a*) Fights won either by decision or defeat (KO/TKO and submission); (*b*) fights won by decision, KO/TKO or submission. Dots represent the raw data, lines show predicted probability of winning based on general linear models (note that random effects of fighter IDs are missing from this prediction), and error bands illustrate 95% confidence intervals for these predictions.

**Figure 2.**
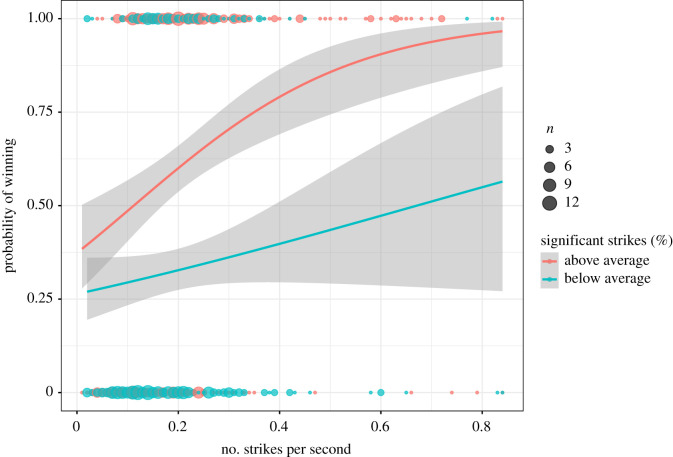
Interaction between per cent significant strikes landed (skill) and the number of strikes attempted per second (vigour) on the likelihood of winning. Here, skill has been split into a dichotomous variable (high, low) to aid visualization of this interaction, where the chance of victory increases with vigour more markedly for the most skilful fighters (red line) compared with the least skilful fighters. Dots represent the raw data, lines show predicted probability of winning based on general linear models (note that random effects of fighter IDs are missing from this prediction), and error bands illustrate 95% confidence intervals for these predictions.

We found a significant interaction between the method of resolution and strikes per second on the focal outcome (*β* = −0.98 ± 0.32, $\chi_{1,9}^{2}=10.297$, *p* = 0.001); in all fights winners fought more vigorously than losers, but this performance trait was more important for fights resolved via the judges' decision compared with those resolved by a defeat (figure 1*a*). Furthermore, there was a significant interaction between the per cent significant strikes and strikes per second on focal outcome (*β* = 0.49 ± 0.15, $\chi_{{{\;}_{1}}_{,8}}^{2}=12.77$, *p* < 0.001), whereby the probability of winning increased with vigour (percentage of strikes per second) and this effect of vigour was enhanced by skill (percentage of significant strikes). In other words, the positive relation between vigour and outcome increases with skill (figure 2). There was no significant effect of sex or its interactions with other fixed effects (see electronic supplementary material for a summary of the model).

### (b) Decision, knockout and submission

When including all three possible methods of resolution in our full model, we found a significant interaction between method and strikes attempted per second ($\chi_{2,9}^{2}=9.66$, *p* = 0.008), again indicating that vigour was most important for success in fights ending via a decision (figure 1*b*). As above, there was also a significant positive interaction between the two performance traits (*β* = 0.50 ± 0.15, $\chi_{1,10}^{2}=13.20$, *p* < 0.001). There was no significant effect of sex or its interactions with other fixed effects (see electronic supplementary material for a summary of the model).

## 4. Discussion

Our results indicate that judges' decisions in MMA fights are, in general, based on the same performance traits that determine actual fighting success (via KO/submission). Thus, in this example of human combat, it appears that an observer's perceptions of the disparity in performance between two opponents, and hence of their relative RHPs, matches their actual abilities. In both analyses, we found that fighting success was determined jointly by skill (% significant strikes landed) and vigour (no. strikes attempted per second), but that vigour was more important for winning by decision than it was for actual fighting success (KO/TKO or submission).

Previous research has shown that humans are able to accurately assess the strength [32] and fighting ability of athletes based solely on the perception of facial cues [33]. However, this accuracy is only observed when the participants can compare the faces of opponents (i.e. comparing relative ‘aggressiveness' of fighters' facial cues) and only when presented with the faces of heavyweight fighters [34]. The results of our study suggest that humans' ability to accurately assess fighting ability extends beyond morphological cues to performance traits that have a direct impact on fighting success. Nevertheless it is important to bear in mind that MMA is a complex sport and as with all contests, animal and human, the determinants of success are likely to be far more complex than the two striking metrics we have measured here. MMA consists of both striking and grappling, and while for the purposes of this study we focused on striking, the question remains as to whether grappling performance is of equal importance for fighting success via perceived or actual defeat.

Our results indicate a positive interaction between the effects of vigour and skill on the likelihood of winning a fight, regardless of the method of resolution. This indicates that striking at a high rate is more effective when the accuracy of the strikes is high. This makes sense as a fighter may attempt a high number of strikes with low skill, missing the target more often than not, and thus fail to accrue either damage on the opponent or points from judges. This interaction also suggests that the benefits gained from landing accurate strikes are enhanced as vigour increases.

MMA fights ending via decision are by definition longer than fights ending by KO or submission, which can be over within seconds. It is therefore unsurprising that our data show vigour to be lower in fights ending via decision (figure 1), as longer fights lead to higher levels of fatigue among fighters. It is interesting to note, however, that there was no such difference in the level of skill exhibited, despite this disparity in fight length, potentially indicating that striking skill is not constrained by fatigue. Although vigour is lower overall in fights ending via decision, the interaction between vigour and the method of resolution indicates that this temporal performance measure is more important for winning via a decision than it is for executing a defeat naturally. There may be several reasons for this. First, as fighters become more fatigued, differences in strike rate between opponents may become more marked, especially if one fighter tires more quickly than the other; thus vigour may become a more informative measure of RHP in the later stages of a fight. However, as MMA fight scores are cumulative, taking into account all rounds, this suggestion seems unlikely. Alternatively, judges may find vigour easier to assess than skill, leading them to overvalue the contribution of vigour to RHP. MMA is a fast-paced sport and it would be interesting to explore whether this mismatch in the value of vigour changes depending on the judges' access to ‘instant replay', a tool now widely used in the sports industry to accurately assess outcomes. Whether bystanders other than official judges (e.g. audience members) also overestimate the contribution of vigour to victory warrants future investigation.

To what extent do these findings support the key assumption of animal contest theory, that RHP assessment should be reasonably accurate? We have shown that human observers can accurately assess RHP, an ability which would benefit animal bystanders that use information on fighters' RHP to inform their choice of future mates [3,11–13] and opponents [14,15]. However, the situation is likely to be different for individuals that are engaged in a fight. The ability to accurately assess an opponent is likely to vary with the escalation patterns of a fight. For instance, accurate assessments are more likely to be made in contests (or phases) characterized by an exchange of signals, in comparison with escalated fights involving direct physical contact and injuries. In this latter case, the accrued costs of competing are likely to have a stronger effect on giving-up decisions. Indeed, studies have shown that individuals switch assessment strategies as the fight progresses, using mutual assessment during the early stages of a fight and switching to self-assessment as the fight escalates [35,36].

Here we have shown that while the same agonistic performance traits are important for victory via decision and actual defeat, these traits are not necessarily of equal importance in both cases, with vigour being overvalued in perceptions of fighting ability. Given the fundamental role of assessment during animal contests, determining whether similar disparities exist between perceived and actual RHP in animal fights should be a priority.

## Supplementary Material

Descriptive statistics and model summaries

## Supplementary Material

R Code for analysis

## Data accessibility

Data for this article are available in the Dryad Digital Repository at https://doi.org/10.5061/dryad.8sf7m0ckf [37].
