# Supplementary material for: Perceived and actual fighting ability: determinants of success by decision, knockout or submission in human combat sports
Source: Biol Lett. 2020 Oct 28;16(10):20200443. doi: 10.1098/rsbl.2020.0443 (PMC7655483; doi:10.1098/rsbl.2020.0443)
Supplement: Descriptive statistics and model summaries [file rsbl20200443supp1.docx]

**Supplementary material for:**

Lane SM & Briffa M. 2020 Perceived and actual fighting ability: Determinants of success via decision, knockout or submission in human combat sports. *Biology Letters.*

**Description of fight data**

- MMA fight data were collated from UFCstats.com (a database which provides the official statistics for the Ultimate Fighting Championship (UFC)) for all completed fights listed from February 2019 – March 2020 (*N* = 548 fights; women’s = 102, men’s = 446).These fights involving 599 different fighters who fought an average of 1.83 times each (range = 1-6 fights per athlete).
- Method of resolution: - Decision = 303; KO/TKO = 160; Submission = 80; Doctor’s stoppage = 5.
- Fight duration: - Decision = were either 900 seconds or 1500 seconds depending on number of rounds fought (3 or 5); KO/TKO = Mean 227.26 seconds (±15.43s); Submission = Mean 260.28 seconds (±15.77s).

**Analysis with only decision/defeat as methods of resolution**

**Summary of fully fitted model showing effect sizes: -**

Generalized linear mixed model fit by maximum likelihood (Laplace Approximation) [glmerMod]

Family: binomial ( logit )

Formula: Outcome ~ Method * ZSig.strikes * ZStrikes_Sec * Sex + (1 | RedID) +

(1 | BlueID)

AIC BIC logLik deviance df.resid

670.6 748.0 -317.3 634.6 525

Scaled residuals:

Min 1Q Median 3Q Max

-12.9180 -0.7209 -0.4083 0.8259 2.7030

Random effects:

Groups Name Variance Std.Dev.

RedID (Intercept) 1.480e-01 0.384684

BlueID (Intercept) 6.212e-06 0.002492

Number of obs: 543, groups: RedID, 396; BlueID, 390

Fixed effects:

Estimate Std. Error z value Pr(>|z|)

(Intercept) 0.37739 0.28610 1.319 0.1871

MethodDefeat -1.02565 0.58750 -1.746 0.0808 .

ZSig.strikes 1.34154 0.53521 2.507 0.0122 *

ZStrikes_Sec 0.72508 0.60727 1.194 0.2325

SexM 0.04680 0.35465 0.132 0.8950

MethodDefeat:ZSig.strikes -0.35314 0.71205 -0.496 0.6199

MethodDefeat:ZStrikes_Sec -0.43707 0.67262 -0.650 0.5158

ZSig.strikes:ZStrikes_Sec 0.91324 0.90695 1.007 0.3140

MethodDefeat:SexM 0.35523 0.64118 0.554 0.5796

ZSig.strikes:SexM -0.03267 0.62871 -0.052 0.9586

ZStrikes_Sec:SexM 0.99261 0.70883 1.400 0.1614

MethodDefeat:ZSig.strikes:ZStrikes_Sec -0.79544 0.95619 -0.832 0.4055

MethodDefeat:ZSig.strikes:SexM -0.20179 0.80763 -0.250 0.8027

MethodDefeat:ZStrikes_Sec:SexM -0.85286 0.78226 -1.090 0.2756

ZSig.strikes:ZStrikes_Sec:SexM -0.04808 1.03225 -0.047 0.9629

MethodDefeat:ZSig.strikes:ZStrikes_Sec:SexM 0.43199 1.09329 0.395 0.6927

---

Signif. codes: 0 ‘***’ 0.001 ‘**’ 0.01 ‘*’ 0.05 ‘.’ 0.1 ‘ ’ 1

**Summary of minimal adequate model: -**

Generalized linear mixed model fit by maximum likelihood (Laplace Approximation) [glmerMod]

Family: binomial ( logit )

Formula: Outcome ~ Method + ZSig.strikes + ZStrikes_Sec + Sex + (1 | RedID) +

(1 | BlueID) + Method:ZStrikes_Sec + ZSig.strikes:ZStrikes_Sec

AIC BIC logLik deviance df.resid

660.8 699.5 -321.4 642.8 534

Scaled residuals:

Min 1Q Median 3Q Max

-12.8607 -0.7246 -0.3976 0.8194 2.3568

Random effects:

Groups Name Variance Std.Dev.

RedID (Intercept) 1.337e-01 0.365615

BlueID (Intercept) 9.702e-06 0.003115

Number of obs: 543, groups: RedID, 396; BlueID, 390

Fixed effects:

Estimate Std. Error z value Pr(>|z|)

(Intercept) 0.24801 0.23789 1.043 0.29716

MethodDefeat -0.60031 0.22656 -2.650 0.00806 **

ZSig.strikes 0.90514 0.13755 6.580 4.69e-11 ***

ZStrikes_Sec 1.37556 0.29148 4.719 2.37e-06 ***

SexM 0.04154 0.26316 0.158 0.87457

MethodDefeat:ZStrikes_Sec -0.97612 0.31761 -3.073 0.00212 **

ZSig.strikes:ZStrikes_Sec 0.48517 0.15088 3.216 0.00130 **

---

Signif. codes: 0 ‘***’ 0.001 ‘**’ 0.01 ‘*’ 0.05 ‘.’ 0.1 ‘ ’ 1

Correlation of Fixed Effects:

(Intr) MthdDf ZSg.st ZStr_S SexM MD:ZS_

MethodDefet -0.267

ZSig.striks 0.109 -0.234

ZStrikes_Sc 0.149 -0.424 0.186

SexM -0.769 -0.224 0.002 0.229

MthdDf:ZS_S -0.166 0.334 -0.133 -0.896 -0.166

ZSg.st:ZS_S 0.126 -0.070 0.480 0.111 -0.072 -0.101

**Analysis with all methods of resolution (Decision; KO/TKO; Submission)**

**Summary of fully fitted model showing effect sizes: -**

Generalized linear mixed model fit by maximum likelihood (Laplace Approximation) ['glmerMod']

Family: binomial ( logit )

Formula: Outcome ~ Method * ZSig.strikes * ZStrikes_Sec * Sex + (1 | RedID) +

(1 | BlueID)

AIC BIC logLik deviance df.resid

674.7 786.4 -311.4 622.7 517

Scaled residuals:

Min 1Q Median 3Q Max

-16.6030 -0.7433 -0.3404 0.8436 2.7717

Random effects:

Groups Name Variance Std.Dev.

RedID (Intercept) 4.767e-02 0.2183380

BlueID (Intercept) 2.169e-08 0.0001473

Number of obs: 543, groups: RedID, 396; BlueID, 390

Fixed effects:

Estimate Std. Error z value Pr(>|z|)

(Intercept) 0.37569 0.27923 1.345 0.1785

MethodKO/TKO -0.82536 0.73628 -1.121 0.2623

MethodSubmission -0.63200 1.06173 -0.595 0.5517

ZSig.strikes 1.30727 0.52392 2.495 0.0126 *

ZStrikes_Sec 0.71228 0.59614 1.195 0.2322

SexM 0.03976 0.34663 0.115 0.9087

MethodKO/TKO:ZSig.strikes -0.56471 0.75631 -0.747 0.4553

MethodSubmission:ZSig.strikes 0.28657 1.17246 0.244 0.8069

MethodKO/TKO:ZStrikes_Sec -0.81070 0.75159 -1.079 0.2807

MethodSubmission:ZStrikes_Sec 0.79307 1.56083 0.508 0.6114

ZSig.strikes:ZStrikes_Sec 0.89558 0.88695 1.010 0.3126

MethodKO/TKO:SexM -0.17567 0.79279 -0.222 0.8246

MethodSubmission:SexM 0.43003 1.10904 0.388 0.6982

ZSig.strikes:SexM -0.02174 0.61709 -0.035 0.9719

ZStrikes_Sec:SexM 0.97082 0.69421 1.398 0.1620

MethodKO/TKO:ZSig.strikes:ZStrikes_Sec -0.42516 0.99748 -0.426 0.6699

MethodSubmission:ZSig.strikes:ZStrikes_Sec -0.29565 1.90935 -0.155 0.8769

MethodKO/TKO:ZSig.strikes:SexM 0.24888 0.85608 0.291 0.7713

MethodSubmission:ZSig.strikes:SexM -1.17906 1.25478 -0.940 0.3474

MethodKO/TKO:ZStrikes_Sec:SexM -0.30697 0.85906 -0.357 0.7208

MethodSubmission:ZStrikes_Sec:SexM -2.17902 1.62215 -1.343 0.1792

ZSig.strikes:ZStrikes_Sec:SexM -0.04694 1.01112 -0.046 0.9630

MethodKO/TKO:ZSig.strikes:ZStrikes_Sec:SexM 0.06247 1.13372 0.055 0.9561

MethodSubmission:ZSig.strikes:ZStrikes_Sec:SexM -0.22400 2.00090 -0.112 0.9109

---

Signif. codes: 0 ‘***’ 0.001 ‘**’ 0.01 ‘*’ 0.05 ‘.’ 0.1 ‘ ’ 1

**Summary of minimal adequate model: -**

Generalized linear mixed model fit by maximum likelihood (Laplace Approximation) ['glmerMod']

Family: binomial ( logit )

Formula: Outcome ~ Method + ZSig.strikes + ZStrikes_Sec + Sex + (1 | RedID) +

(1 | BlueID) + Method:ZStrikes_Sec + ZSig.strikes:ZStrikes_Sec

AIC BIC logLik deviance df.resid

658.6 705.9 -318.3 636.6 532

Scaled residuals:

Min 1Q Median 3Q Max

-12.3972 -0.7249 -0.4001 0.8258 2.3888

Random effects:

Groups Name Variance Std.Dev.

RedID (Intercept) 7.796e-02 0.279212

BlueID (Intercept) 2.325e-06 0.001525

Number of obs: 543, groups: RedID, 396; BlueID, 390

Fixed effects:

Estimate Std. Error z value Pr(>|z|)

(Intercept) 0.2468 0.2353 1.049 0.294232

MethodKO/TKO -0.8914 0.2607 -3.420 0.000627 ***

MethodSubmission -0.1068 0.2970 -0.360 0.719141

ZSig.strikes 0.9287 0.1391 6.678 2.42e-11 ***

ZStrikes_Sec 1.3711 0.2883 4.755 1.99e-06 ***

SexM 0.0489 0.2603 0.188 0.850993

MethodKO/TKO:ZStrikes_Sec -0.9652 0.3354 -2.878 0.004002 **

MethodSubmission:ZStrikes_Sec -0.8959 0.3738 -2.396 0.016556 *

ZSig.strikes:ZStrikes_Sec 0.5024 0.1546 3.251 0.001151 **

---

Signif. codes: 0 ‘***’ 0.001 ‘**’ 0.01 ‘*’ 0.05 ‘.’ 0.1 ‘ ’ 1

Correlation of Fixed Effects:

(Intr) MtKO/TKO MthdSb ZSg.st ZStr_S SexM MKO/TKO: MS:ZS_

MethdKO/TKO -0.229

MethdSbmssn -0.207 0.340

ZSig.striks 0.118 -0.276 -0.082

ZStrikes_Sc 0.151 -0.382 -0.298 0.190

SexM -0.769 -0.206 -0.148 -0.003 0.228

MKO/TKO:ZS_ -0.176 0.235 0.244 -0.158 -0.844 -0.138

MthdSb:ZS_S -0.122 0.276 0.252 -0.078 -0.751 -0.157 0.628

ZSg.st:ZS_S 0.137 -0.086 -0.010 0.493 0.112 -0.081 -0.176 0.019
